# Parathyroid Carcinoma: A Clinical Case Report and Literature Review

**DOI:** 10.7759/cureus.89550

**Published:** 2025-08-07

**Authors:** Mohamed Afellah, Anouar Ben Ameur El Youbi, Sanae Chahbouni, Naouar Ouattassi, Mohammed Ridal, Najib Benmansour, Zouheir Zaki, Abdellatif Oudidi

**Affiliations:** 1 Department of ENT, University Hospital Center Hassan II, Fez, MAR; 2 Anatomical Pathology, Shifa Laboratory, Fez, MAR

**Keywords:** hypercalcemia, neck mass, parathyroid carcinoma, primary hyperparathyroidism, rare tumor

## Abstract

Parathyroid carcinoma is an exceptionally rare malignant tumor of the parathyroid gland. Clinically, it most often presents as severe primary hyperparathyroidism. Diagnosis relies on histopathological analysis, although it is often difficult to establish due to the lack of clearly pathognomonic criteria. Management is primarily based on complete surgical excision. We report the case of a 55-year-old woman with no significant medical history, admitted for evaluation of a suspicious parathyroid nodule discovered in the context of hyperparathyroidism. She underwent parathyroidectomy along with total thyroidectomy. The postoperative course was favorable, with normalization of laboratory parameters and no signs of recurrence. This case highlights the diagnostic complexity of parathyroid carcinoma, despite suggestive clinical, biological, and imaging findings. Histological diagnosis remains challenging due to overlapping features with benign lesions. Complete surgical excision during the initial operation is the main favorable prognostic factor, both for therapeutic success and prevention of recurrence.

## Introduction

Parathyroid carcinoma is an exceptionally rare neoplasm of the parathyroid glands, accounting for fewer than 1% of all cases of primary hyperparathyroidism [1]. Its clinical presentation is often dominated by complications of severe hypercalcemia, such as nephrolithiasis, bone disease, or neuropsychiatric symptoms [2]. Despite suggestive clinical and biochemical features, establishing a definitive diagnosis remains challenging, as histopathological criteria may be inconclusive and overlap with benign lesions. Optimal prognosis depends on complete surgical excision during the initial operation [2]. This article aims to illustrate the diagnostic challenges and therapeutic considerations associated with parathyroid carcinoma, supplemented by a review of the current literature.

## Case presentation

A 55-year-old woman with no significant past medical history presented to our department with a progressively enlarging anterior cervical mass. In addition to this cervical swelling, the patient reported diffuse bone pain in the context of general health deterioration. She did not report any dysphagia, dyspnea, or dysphonia.

On clinical examination, there was a firm, non-tender, well-delimited mass located in the lower left anterior cervical region, mobile with swallowing, consistent with a thyroid-related lesion. No overlying skin changes or local inflammatory signs were observed. There was no palpable cervical lymphadenopathy, and the rest of the physical examination was unremarkable.

Routine laboratory investigations revealed: (1) marked hypercalcemia: 121 mg/L (reference range: 85-105 mg/L); significantly elevated parathyroid hormone (PTH): 385 pg/mL (reference range: 15-65 pg/mL), representing nearly six times the upper normal limit; and elevated alkaline phosphatase: 1,230 IU/L (reference range: 45-129 IU/L).

A cervical ultrasound was performed, which demonstrated a predominantly solid, hypoechoic nodule in the lower left pole of the thyroid gland, 11 mm in size, classified as TIRADS V, raising concern for malignancy. Two additional mixed, predominantly solid nodules were noted in the mid and lower poles of the right thyroid lobe, measuring 11 mm and 13 mm, respectively, both classified as TIRADS IV. Moreover, a retrothyroidal, oval-shaped, homogeneously hypoechoic and markedly hypervascular nodule was identified in the upper left pole, measuring 23 × 9 mm, suggestive of a parathyroid origin.

**Figure 1:**
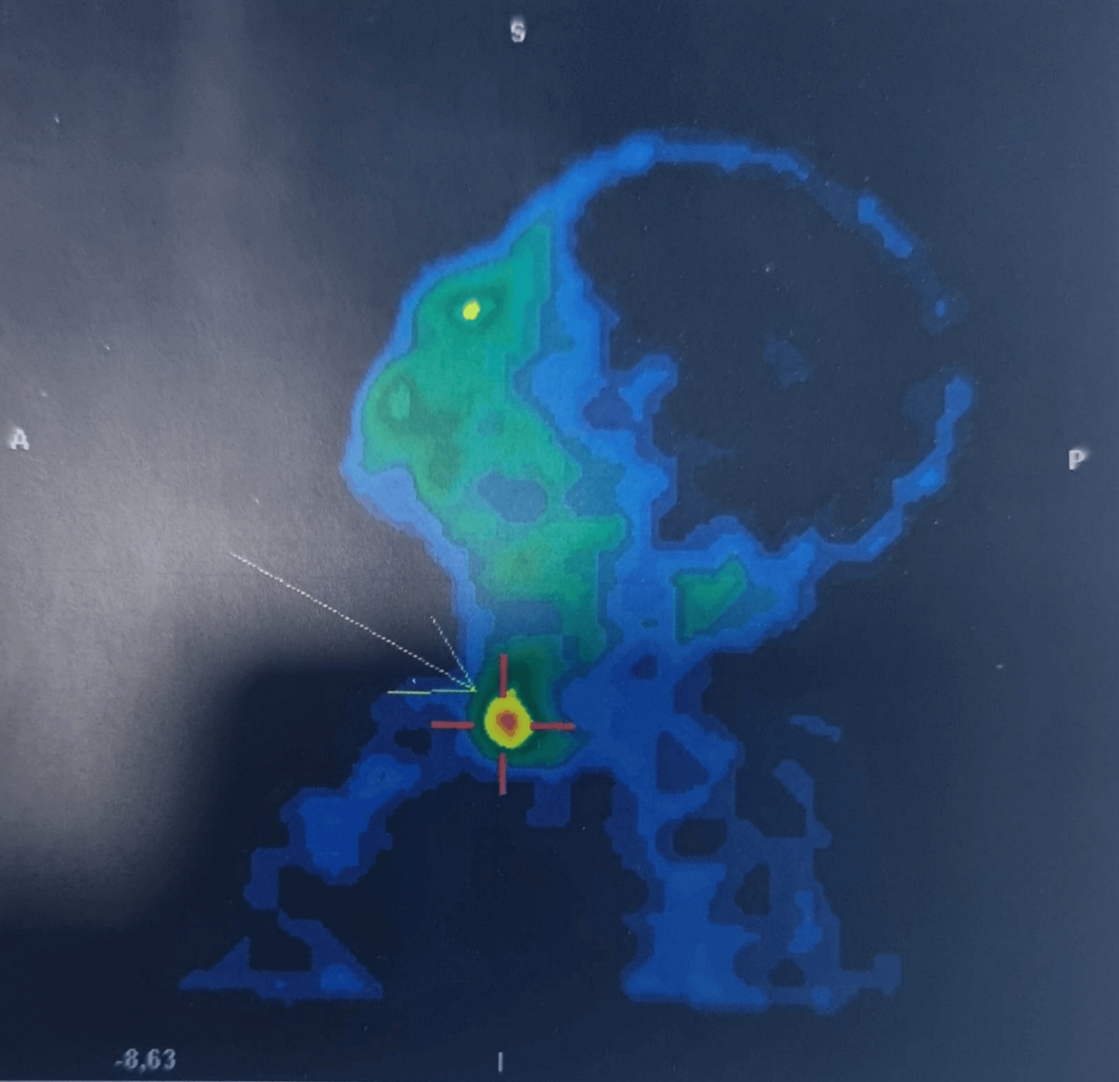
Parathyroid scintigraphy showing focal hyperfixation consistent with a left inferior parathyroid nodule (arrow).

To further characterize the suspicious lesion, a technetium-99m sestamibi scintigraphy fused with computed tomography (SPECT/CT) was performed. It confirmed focal radiotracer uptake corresponding to the retrothyroidal nodule described above, consistent with a hyperfunctioning parathyroid adenoma or carcinoma (Figure 1).

Based on the biochemical and imaging findings, the patient underwent surgical treatment including a total thyroidectomy with en bloc resection of the left inferior parathyroid gland.

The postoperative course was uneventful, with no immediate surgical complications. A rapid and sustained normalization of serum calcium and PTH levels was observed, indicating successful removal of the hyperfunctioning parathyroid tissue.

**Figure 2:**
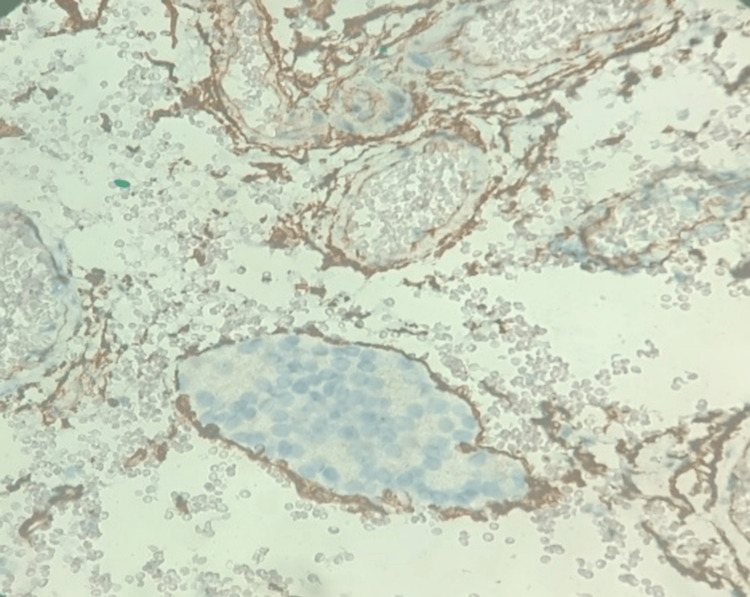
Immunohistochemical analysis using CD34 highlights the presence of a tumor embolus within an extracapsular compartment.

Histological analysis of the excised parathyroid lesion revealed features consistent with parathyroid carcinoma, including cellular proliferation with atypical mitoses, and evidence of vascular invasion, as demonstrated by the presence of CD34-positive vascular emboli (Figure 2). These criteria, especially vascular invasion, are among the few recognized histological features suggestive of malignancy in parathyroid tumors.

Histological examination of the thyroid specimen revealed a multinodular hyperplastic goiter accompanied by lymphocytic thyroiditis, with no histological evidence of malignancy within the thyroid nodules.

At the one-year follow-up, the patient remained clinically asymptomatic, with normal serum calcium and PTH levels, and no evidence of local or distant recurrence on follow-up imaging. This favorable evolution highlights the importance of early diagnosis and complete surgical excision in managing parathyroid carcinoma.

## Discussion

Parathyroid carcinoma is an extremely rare malignant neoplasm, accounting for fewer than 0.005% of all cancers [1]. Its incidence is estimated at 4-6 cases per year per 10 million people [1]. It typically occurs between the ages of 45 and 59 years, with no sex predilection, unlike parathyroid adenomas, which are more common in women [2].

The etiology of parathyroid carcinoma remains unknown, although complex interactions between environmental and genetic factors are suspected. Early exposure to radiotherapy may increase the risk of parathyroid pathology, though no formal link to carcinoma has been established [3,4]. It usually occurs sporadically but may also present in hereditary contexts, particularly in familial hyperparathyroidism and multiple endocrine neoplasia (MEN) syndromes types 1 and 2A [5].

Differentiating between benign and malignant parathyroid tumors remains a clinical challenge. Parathyroid carcinoma most commonly presents with severe primary hyperparathyroidism, leading to significant hypercalcemia [6]. Certain clinical features may raise suspicion of malignancy; notably, a palpable neck mass is observed in approximately 65% of cases [7]. Biochemically, serum calcium and PTH levels are significantly higher than in benign conditions. Elevated levels of alkaline phosphatase may also be observed [6].

On cervical ultrasound, several features suggest malignancy: size greater than 2 cm, hypoechogenicity, irregular margins, lobulations, evidence of local invasion, or suspicious cervical lymphadenopathy. However, none of these features is specific to parathyroid carcinoma [7]. Technetium-99m sestamibi scintigraphy is essential for diagnostic guidance, with a sensitivity of up to 91%, facilitating the localization of hyperfunctioning parathyroid lesions [8]. Thoraco-abdominopelvic CT is recommended to screen for distant metastases [1]. Lymph node metastases, and especially pulmonary and bone metastases, are present in 30% of parathyroid carcinoma cases [9].

The diagnosis of parathyroid carcinoma remains challenging, as no single histopathological criterion is pathognomonic. Features such as capsular or vascular invasion, although suggestive of malignancy, are inconsistently observed and lack sensitivity [5]. The current World Health Organization classification relies on a combination of minor criteria (capsular or soft tissue invasion) and major criteria (vascular invasion or presence of metastases) [5,10]. Immunohistochemistry, particularly the loss of parafibromin expression, has emerged as a valuable tool to distinguish carcinoma from benign lesions [11]. Loss of parafibromin also carries prognostic significance, being associated with higher recurrence rates and reduced overall survival [12].

The gold standard treatment is complete surgical resection, including removal of the pathological parathyroid gland and the ipsilateral thyroid lobe [6]. Adjuvant radiotherapy may improve local disease control and reduce recurrence, prolonging remission regardless of the initial surgical approach. In contrast, adjuvant chemotherapy has no current indication, and polychemotherapy regimens used in metastatic cases have not shown a survival benefit [7].

Recurrence rates, whether local or distant, range from 25% to 60% within two to five years following initial surgery. While most relapses occur within the first three years, late recurrences have been reported, sometimes beyond two decades, justifying prolonged and rigorous surveillance [1].

## Conclusions

Parathyroid carcinoma is a rare tumor with a challenging diagnosis, despite suggestive clinical, biochemical, and histological findings. Surgery remains the mainstay of treatment, with a better prognosis when performed early and completely. Long-term follow-up is essential due to the high risk of recurrence, which may occur even decades later.
